# Intranasal Galanin (1–15) modulates alcohol self-administration and depressive-like behavior in rats and shows a favorable safety profile

**DOI:** 10.3389/fphar.2025.1719440

**Published:** 2025-11-26

**Authors:** Noelia Cantero-García, Antonio Flores-Burgess, Marta Flores-Gómez, Juan Pedro Pineda-Gómez, Maria C. Ramos, Caridad Diaz, Carmelo Millón, Zaida Díaz-Cabiale

**Affiliations:** 1 Universidad de Málaga, Instituto de Investigación Biomédica de Málaga, Facultad de Medicina, Málaga, Spain; 2 Fundación MEDINA, Granada, Spain

**Keywords:** galanin, intranasal, safety profile, depression, alcohol disorders

## Abstract

**Introduction:**

Galanin is a neuropeptide in the central nervous system that regulates mood and alcohol consumption. Its N-terminal fragment, Galanin (1–15) [GAL (1–15)], has demonstrated specific behavioral effects, enhancing the efficacy of selective serotonin reuptake inhibitors (SSRIs) and reducing ethanol intake and alcohol self-administration in preclinical models. Additionally, GAL (1–15) combined with SSRIs decreases alcohol self-administration and depressive symptoms in a comorbid alcoholism-depression model. These findings suggest potential applications in the treatment of depression and alcohol use disorders. Given its therapeutic potential, evaluating the translational aspects and safety of GAL (1–15) is essential for clinical development.

**Methods:**

This study investigated the intranasal administration of GAL (1–15), assessing its effects on depression-related behavior in the Forced Swim Test (FST) and alcohol self-administraation in an operant selfadministration model. To determine its safety profile, we evaluated GAL (1–15) for cardio toxicity (hERG channel inhibition), genotoxicity (micronucleus formation), and drug interactions (CYP450 inhibition).

**Results:**

Results showed that intranasal GAL (1–15) exhibited a prodepressant effect, as previously demonstrated with its ICV administration. Furthermore, intranasal GAL (1–15) effectively reduced alcohol consumption. In vitro safety assessments revealed no adverse effects on cardiac function or genotoxicity at the concentrations studied, with minimal interaction with CYP450 enzymes, supporting its suitability for drug development.

**Discussion:**

In conclusion, intranasal administration of GAL (1–15) represents a promising candidate for clinical trials due to its favorable safety profile and therapeutic potential. Its use appears particularly suitable as a standalone treatment for alcohol use disorder and, when combined with SSRIs, as a potential therapy for depressive disorders.

## 1 Introduction

Galanin (GAL) is a 29-amino acid neuropeptide with a highly conserved N-terminal sequence across species (Tatemoto et al., 1983; Wiczk et al., 1996). It is widely distributed in the central nervous system (CNS), particularly in the cerebral cortex, hippocampus, and brainstem. GAL is synthesized in the noradrenergic neurons of the locus coeruleus (LC) and serotonergic neurons of the dorsal raphe nucleus (DR) (Jacobowitz et al., 2004; Skofitsch and Jacobowitz, 1985; Melander et al., 1986).

Functionally, GAL is involved in various physiological processes, including the central regulation of food intake, alcohol consumption, cognitive performance, cardiovascular function, energy homeostasis, neuroendocrine regulation, and mood regulation (Mitsukawa et al., 2008; Díaz-Cabiale et al., 2010; Fang et al., 2015; Lang et al., 2007). GAL mediates its biological effects through 3 G protein-coupled receptors (GALR1-3) (Branchek et al., 2000). GALR1 and GALR3 predominantly activate inhibitory G proteins (Gi/o), whereas GALR2 primarily couples with Gq/11, triggering excitatory signalling (Lang et al., 2007). These receptors are widely distributed in different brain regions, often co-localizing (Jacobowitz et al., 2004).

Beyond GAL itself, its N-terminal fragment, GAL (1–15), exhibits distinct biological activity and mechanisms of action (Díaz-Cabiale et al., 2010; Ihnatko and Theodorsson, 2017). GAL (1–15) plays a significant role in cardiovascular regulation (Díaz-Cabiale et al., 2010; Díaz-Cabiale et al., 2005), mood modulation (Millón et al., 2014; Millón et al., 2019a), and alcohol intake control (Millón et al., 2019b; Cantero-García et al., 2022a; Cantero-García et al., 2022b).

Regarding mood regulation, GAL (1–15) has been implicated in mood disorders acting via GALR1-GALR2 heteroreceptor complexes (Millón et al., 2014; Millón et al., 2019a; Borroto-Escuela et al., 2014). GAL (1–15) administration induces depressive and anxiogenic-like effects while enhancing the antidepressant efficacy of the 5-HT1A receptor (5-HT1AR) agonist 8-OH-DPAT in the Forced Swim Test (FST) (Millón et al., 2016). This mechanism involves alterations in 5-HT1AR binding and mRNA levels in the dorsal hippocampus and DR.

The potential of GAL (1–15) as an adjunct to selective serotonin reuptake inhibitors (SSRIs) was explored, showing that GAL (1–15) potentiated fluoxetine (FLX) antidepressant effects and mitigated memory impairment, involving 5-HT1AR in the hippocampus and prefrontal cortex (PFC) (Flores-Burgess et al., 2017; Flores-Burgess et al., 2019). These results led to an international PCT patent application entitled “Pharmaceutical composition comprising SSRI and GAL (1–15)” (WO 2018/150073 A1). Further studies in an olfactory bulbectomy (OBX) depression model revealed that GAL (1–15) enhanced FLX’s antidepressant-like effects in despair and anhedonia tests through 5-HT1AR modulation at the plasma membrane and transcriptional levels (Flores-Burgess et al., 2022). GAL (1–15) also augmented escitalopram’s antidepressant effects in OBX rats via 5-HT1AR activation, an effect abolished by 5-HT1AR downregulation via siRNA (García-Durán et al., 2021). Two significant networks mediate GAL (1–15) modulation of escitalopram activity: one involving the lateral (LHb) and medial (mHb) habenula, DR, and ventral tegmental area (VTA), and another comprising the dentate gyrus (DG) and PFC (García-Durán et al., 2021).

Moreover, we recently described that GAL (1–15) significantly enhanced FLX’s antidepressant-like effects, particularly in despair-related behaviors in the Wistar-Kyoto (WKY) rat model (Pineda-Gómez et al., 2025). This model mimics endogenous depression with alterations in monoamine, glutamate, and GABA systems, as well as hypothalamic-pituitary-adrenal (HPA) axis dysregulation. Because standard antidepressants, especially SSRIs, show limited efficacy in WKY rats, this strain is regarded as a useful model for studying treatment-resistant depression (TRD). TRD is a prevalent disorder that affects up to 30% of patients with major depressive disorder and is associated with a significant functional and economic burden (Fiorillo et al., 2025). GALR2 involvement was suggested by M871, a GALR2 antagonist, blocking GAL (1–15)’s effects in the FST. Additionally, siRNA-mediated 5-HT1AR downregulation abolished GAL’s enhancement of FLX’s effects (1–15) (Pineda-Gómez et al., 2025). The combination of GAL (1–15) and FLX, but not FLX alone, normalized the abnormally high corticosterone levels in WKY rats, aligning with findings in the OBX depression model, suggesting GAL (1–15) may regulate HPA axis activity (Pineda-Gómez et al., 2025).

Regarding the role of GAL in Alcohol Use Disorder (AUD), our research has demonstrated that GAL (1–15) significantly reduces ethanol preference and consumption in rats via central mechanisms, particularly in the striatum, a critical region in drug reward and motivation (Millón et al., 2019b). Furthermore, intracerebroventricular (icv) administration of GAL (1–15) reduces alcohol self-administration in an operant self-administration model and prevents context-induced relapse (Cantero-García et al., 2022a). This effect involves the mesocorticolimbic pathway, a key network in drug reward (Cantero-García et al., 2022a). The findings led to an international patent related to GAL (1–15) and its analogues for the treatment of alcohol-related disorders (WO 2019/068948 A1). These findings provide further evidence for the role of GAL (1–15) in alcohol motivation and its potential as a pharmacological treatment for alcohol use disorder (AUD).

Additionally, combining GAL (1–15) with the antidepressant escitalopram effectively reduces alcohol self-administration in operant models, presenting a novel therapeutic approach for alcoholism-depression comorbidity (Cantero-García et al., 2022b).

Given the promising preclinical findings on GAL (1–15), it is essential to address key translational aspects to facilitate its potential clinical application.

A primary consideration is the route of administration, as all previous studies have relied on intracerebroventricular delivery. Therefore, investigating the feasibility of an administration route compatible with patient use is a crucial step.

A variety of substances, including neuropeptides, have been shown to cross the nose-to-brain barrier in both animals and humans (Dufes et al., 2003; Dhuria et al., 2009; Born et al., 2002). Neuropeptides such as NPY and NPS are administered via intranasal (IN) infusion (Serova et al., 2014; Serova et al., 2013; Serova et al., 2019; Ionescu et al., 2012). This approach allows drugs to rapidly and directly reach the central nervous system through intracellular neuronal olfactory and extracellular trigeminal-associated pathways, bypassing the blood-brain barrier and influencing multiple brain regions (Ionescu et al., 2012; Thorne et al., 2004; Thorne et al., 1995; Dhuria et al., 2010), making it a suitable route for neuropeptide administration into the central nervous system.

To date, GAL (1–15) has been administered exclusively via the intracerebroventricular route in animal models. Therefore, it is crucial to assess the effects of this peptide through intranasal administration to enable its effective translation into clinical practice in humans. In addition, evaluating its preliminary safety profile, particularly regarding potential toxicity, is essential to ensure the safe development of its prospective therapeutic applications.

In this study, we examined the effects of intranasal GAL (1–15) on depression-related behavior using the FST and alcohol self-administration in an operant self-administration model. To assess potential cardiotoxicity, we evaluated the inhibition of the human ether-à-go-go-related gene (hERG) potassium channel, a key determinant of drug-induced cardiac arrhythmias. Genotoxicity was evaluated using the *in vitro* Micronucleus Test (MNT), a widely accepted assay for detecting chromosomal damage and genotoxic effects in cultured cells. Finally, we investigated the potential for drug-drug interactions by evaluating GAL (1–15)-mediated inhibition of cytochrome P450 (CYP450) enzymes using a standardized CYP450 inhibition assay.

## 2 Materials and methods

### 2.1 Animals

Male Sprague Dawley rats (body weight 200–225 g) were obtained from Charles River (Barcelona, Spain) and maintained in a humidity-controlled and temperature-controlled (20 °C–22 °C) room. Rats were maintained during the alcohol protocol on a 12-h reversed light/dark cycle (lights off at 9 a.m.). All animal experimentation was conducted in accordance with the University of Málaga Guidelines for the Care and Use of Laboratory Animals (Ethic Code: 22/05/2017/066).

### 2.2 Administration of substances and drugs

Animals were preacclimated to the procedure and handled daily for 3 days to reduce stress. Rats were administered under light anaesthesia with isoflurane (3%–4% for induction and 1.5%–2% for maintenance) for approximately 2–3 min, sufficient to prevent movement during intranasal administration. A single intranasal infusion of GAL (1–15) (TOCRIS, Bristol, United Kingdom, molecular weight = 1557 g/mol) was administered at doses of 37.5 μg, 75 μg, 150 μg, or 300 μg, equivalent to those used with other peptides (Serova et al., 2013; Serova et al., 2019). Doses were dissolved in 20 μL of distilled water, with 10 μL administered into each nostril using a disposable plastic-tipped pipette, without inserting deeply, 30 min or 1 h before behavioral testing, as described previously (Serova et al., 2013; Serova et al., 2019). Care was taken to avoid contact with the intranasal mucosa. After intranasal administration, the animal’s head was held back for approximately 15 s to prevent loss of solution from the nostrils. Animals returned to normal behavior within 1–2 min, and no subjects needed to be withdrawn due to intolerance.

### 2.3 Behavioral assessment

#### 2.3.1 Depressive behavior: forced swimming test

Depressive behavior was assessed using the FST, as described previously (Millón et al., 2014; Cantero-García et al., 2022b). Briefly, two swimming sessions were conducted: a 15-min pretest followed 24 h later by a 5-min test. Animals were individually placed in a vertical glass cylinder of 20 cm diameter containing water (25 °C) to a height of 30 cm. Immobility, swimming and climbing behaviors were evaluated and recorded during the second 5-min test. Behavioral scoring was conducted by a blinded experimenter using coded videos to ensure objectivity.

**FIGURE 1:**
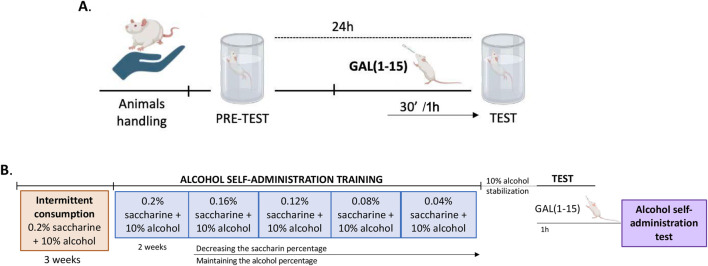
Experimental schedule diagram. **(A)** The effects of different intranasal GAL (1–15) treatments in the forced swimming test (FST) were evaluated in different groups of rats. **(B)** The Effect of the intranasal GAL (1–15) 75 µg administration or distilled water on alcohol self-administration test was analyzed.

Two experiments were conducted in the FST (Figure 1A). A dose-response curve of GAL (1–15) was performed in the first set of experiments. For this, groups of rats received GAL (1–15) intranasally 37.5 μg (n = 6), 75 μg (n = 6), 150 μg (n = 6) or 300 μg (n = 6) or distilled water (n = 12) 30 min before the test. The effects in the FST of GAL (1–15) intranasally at 1 h were studied in the second set of experiments. For this, groups of rats received GAL (1–15) intranasal 37.5 μg (n = 8), 75 μg (n = 8) or distilled water (n = 4–5) 1 h before the test. Animals were randomly assigned to treatment groups.

#### 2.3.2 Alcohol self-administration

Training and testing were conducted in standard operant chambers (Panlab, Barcelona, Spain). Each chamber (250 mm W × 250 mm D × 250 mm H) was equipped with a drinking reservoir positioned 4 cm above the grid floor in the center of the front panel of the chamber, and two retractable levers were located 3 cm to right an left of the dinking receptacle. Each reservoir was calibrated to dispense 0.1 mL when the fixed ratio of lever presses was obtained. Chambers were connected to a computer running Packwin Sofware (2.0, Panlab, Barcelona, Spain).

Alcohol consumption was assessed using the previous self-administration test (Cantero-García et al., 2022a; Cantero-García et al., 2022b; Lebourgeois et al., 2018) (Figure 1B). Briefly, rats were exposed to intermittent consumption of 0.2% saccharin and 10% alcohol for 3 weeks. After that, rats were placed on a water restriction schedule for 2–4 days to facilitate training in lever pressing. The rats were trained to self-administer saccharin 0.2% (w/v) and 10% alcohol (v/v) in 30-min daily sessions for 2 weeks on a fixed ratio 1 schedule of reinforcement in which each response resulted in the delivery of 0.1 mL of fluid. At this point, the saccharin percentage is decreased over the days, while the alcohol concentration is maintained until a stable 10% alcohol response is reached: 0.16% + 0.2% saccharin (3 days), 0.12% + 0.2% saccharin (3 days), 0.08% + 0.2% saccharin (3 days), 0.04% + 0.2% saccharin (4 days), 0.02% + 0.2% saccharin (5 days), stabilization with 10% alcohol (7 days).

During the test sessions, the active and inactive lever responses and the number of alcohol reinforcements were recorded. For this, groups of rats received GAL (1–15) intranasally 75 μg (n = 9) or distilled water (n = 10) 1 h before the alcohol self-administration test. Animals were randomly assigned to treatment groups.

### 2.4 Toxicological analysis

#### 2.4.1 *In vitro* cardiotoxicity

For *in vitro* studies, the GAL (1–15) concentrations used were selected based on previous studies and parameters commonly used in this type of testing, including absorption, distribution, metabolism, excretion (ADME), and preclinical toxicity (Lebourgeois et al., 2018; Nair and Weiskirchen, 2024), so that they reflect levels equivalent to those observed *in vivo* and allow their effects to be related and classified before animal testing. The objective was to choose concentration ranges that would allow adverse effects to be anticipated while also facilitating comparison with concentrations equivalent to those the drug would reach *in vivo*, so that the results could be related to its potential physiological effect.

Cardiotoxicity of GAL (1–15) was assessed in hERG-expressing HEK cells using the FluxOR™ potassium assay (Invitrogen) on a FLIPR TETRA system. Cells were preloaded with FluxOR™ dye, followed by incubation with GAL (1–15) in a 10-point half-log dose-response curve (max concentration: 50 µM). After a 30-min equilibration, the thallium stimulation buffer was injected to measure potassium flux kinetics. Astemizole (10 µM) served as a positive control, and vehicle as a negative control. Data were analyzed using Genedata Screener software, with IC50 values determined via the Hill model (Annang et al., 2021; Lee et al., 2019).

#### 2.4.2 Genotoxicity: *In Vitro* cell Micronucleus Test

The study followed OECD Test Guideline 487 for micronucleus assessment in rodent CHO cells (ECACC ref: 85050302). Cells were seeded at 2,000/well in 96-well plates and incubated at 37 °C with 5% CO_2_. GAL (1–15) (2.52 mM stock) was tested at concentrations ranging from 3.12 to 50 μM for 24 h in six replicates. Mitomycin C served as a positive control, while untreated cells were the negative control. After treatment, cytochalasin B was added for 28 h to block cytokinesis, followed by fixation and Hoechst staining. Imaging was performed using the Operetta CLS High-Content Analysis System, and analysis was conducted with Harmony software and the in-house NucleusFinder App (ImageJ-based). NucleusFinder identified valid micronuclei under stringent criteria, excluding irregular structures (Durcik et al., 2023). The cytokinesis-block proliferation index (CBPI) was calculated to assess cytostatic effects, ensuring cytostasis remained below 60% to avoid secondary micronuclei induction due to cytotoxicity. Cytostasis was calculated as follows: %cytostasis = 100–100 [(CBPIT-1)/(CBPIC-1)] where T is the test compound treatment culture, C is the vehicle control culture, and CBPI = {(No. mononucleate cells) + (2 × No. binucleate cells) + (3 × No. multinucleate cells)}/(total number of cells).

#### 2.4.3 Cytochrome P450 inhibition assay (drug-drug interaction)

The Cytochrome P450 (CYP450) inhibition assay was performed to assess GAL (1–15) using substrates specific for the CYP3A4, CYP2D6, and CYP2C9 isoforms. Test compounds (2.52 mM stock) were serially diluted (0.05–26 μM) and pre-incubated at 37 °C for 10 min with a cofactor/buffer solution (pH 7.4 phosphate buffer, 100 mM; NADPH, 1 mM). Following the addition of human liver microsomes (HLM) and specific substrates (testosterone for CYP3A4, dextromethorphan for CYP2D6, diclofenac for CYP2C9), reactions were incubated with shaking at 180 Hz (CYP3A4: 15 min; CYP2D6: 30 min; CYP2C9: 45 min). Controls included specific inhibitors (ketoconazole for CYP3A4, quinidine for CYP2D6, sulfaphenazole for CYP2C9). Reactions were quenched with acetonitrile, centrifuged, and analyzed using LC-MS/MS with a triple quadrupole mass spectrometer (AB SCIEX API4000) coupled to an Agilent 1290 HPLC system (Fowler et al., 2022). Data were processed using Genedata Screener software to calculate IC50 values.

### 2.5 Statistical analysis

Data are presented as mean ± SEM, with sample sizes (n) indicated in figure legends. Statistical analysis was performed using GraphPad PRISM 8.0. For comparisons between two groups, Student’s unpaired t-tests were employed. One-tailed tests were specifically used in instances where a clear directional hypothesis was established *a priori*, based on previous findings showing that intracerebroventricular administration of GAL (1–15) increases immobility and decreases climbing behavior in the forced swim test. One-way ANOVA followed by Fisher’s LSD *post hoc* test was applied for multiple-group comparisons when the ANOVA F-ratio was significant. Statistical significance was set at p < 0.05 (*p < 0.05, **p < 0.01, ***p < 0.001).

## 3 Results

### 3.1 Behavioral effects

#### 3.1.1 Intranasal administration of GAL (1–15) induces depression-related behavior in the FST

GAL (1–15) was administered intranasally at different doses, and its effects were analyzed at 30 min and 1 h post-administration in the FST.

##### 3.1.1.1 Dose-response effects at 30 minutes post-administration

**FIGURE 2:**
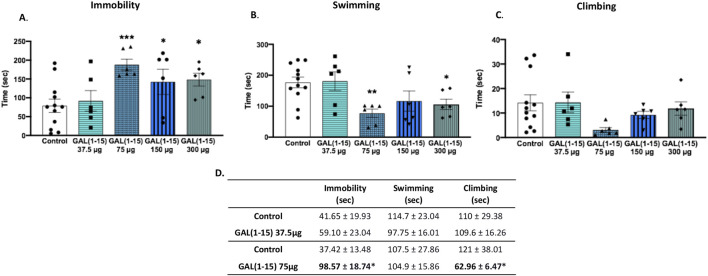
Effect of the intranasal administration of Galanin (1–15) [GAL (1–15)] in the forced swimming test (FST). GAL (1–15) 37 µg (n = 6), 75 µg (n = 6), 150 µg (n = 6), and 300 µg (n = 6), or distilled water (n = 12) was administered intranasally 30 min **(A–C)** or 1 h **(D)** before the test. Rats received 10 µL of substance into each nostril. Distilled water injected rats were used as the control group. **(A–C)** Vertical bars represents the mean ± standard error of the mean immobility, swimming and climbing time. *p < 0.05, **p < 0.01; ***p < 0.001 vs. control group, according to a one-way analysis of variance (ANOVA) between the experimental groups. **(D)** GAL (1–15) 37 µg (n = 8), 75 µg (n = 8), Distilled water (n = 4–5). Data represents mean ± standard error mean of the mean immobility, swimming and climbing time. *p < 0.05 vs. control group, according to a Student’s t-test.

The dose-response curve obtained 30 min after intranasal administration of GAL (1–15) revealed a inverted U-shaped dose–response pattern with significant increase in immobility time at the 75 μg dose compared to control animals (one-way ANOVA, F_4,31_ = 4.19, p = 0.008; Fisher’s LSD *post hoc*: p < 0.001; Figure 2A). Furthermore, this dose significantly decreased swimming time (one-way ANOVA, F_4,31_ = 4.12, p = 0.009; Fisher’s LSD *post hoc*: p < 0.01; Figure 2B). Interestingly, the higher doses of GAL (1–15) (150 μg and 300 μg) also produced a significant increase in immobility and a reduction in swimming time, maintaining the effect observed with the dose of 75 μg (p < 0.05 for both immobility and swimming time). Conversely, the lowest dose tested (37.5 μg) did not produce significant behavioral effects in the FST. No effects were observed from any of the tested doses on climbing time (Figure 2C).

##### 3.1.1.2 Prolonged behavioral effects at 1 hour post-administration

To determine whether GAL (1–15) exerts prolonged effects on depressive-like behavior and to exclude any impact due to the light anaesthesia with isoflurane, the most effective dose (75 μg) and the subthreshold dose (37.5 μg) were administered 1 hour before the FST. Consistent with the earlier findings, the 37.5 μg dose remained ineffective, with no significant alterations in immobility, swimming, or climbing behaviors (Figure 2D). However, the 75 μg dose continued to exert a significant effect, increasing immobility time (Student’s t-test, t_10_ = 2.44, p < 0.05) and reducing climbing time (Student’s t-test, t_10_ = 1.79, p ≤ 0.05). This pattern of effects mirrored those observed following intracerebroventricular (ICV) administration of GAL (1–15), suggesting that the behavioral depression-like impact of GAL (1–15) persists over time and is comparable across different routes of administration (Figure 2D).

#### 3.1.2 Intranasal administration of GAL (1–15) reduces alcohol self-administration in an operant self-administration model

GAL (1–15) was administered intranasally at a dose of 75 μg, and its impact on alcohol reinforcement and lever-pressing behavior was examined.

**FIGURE 3:**
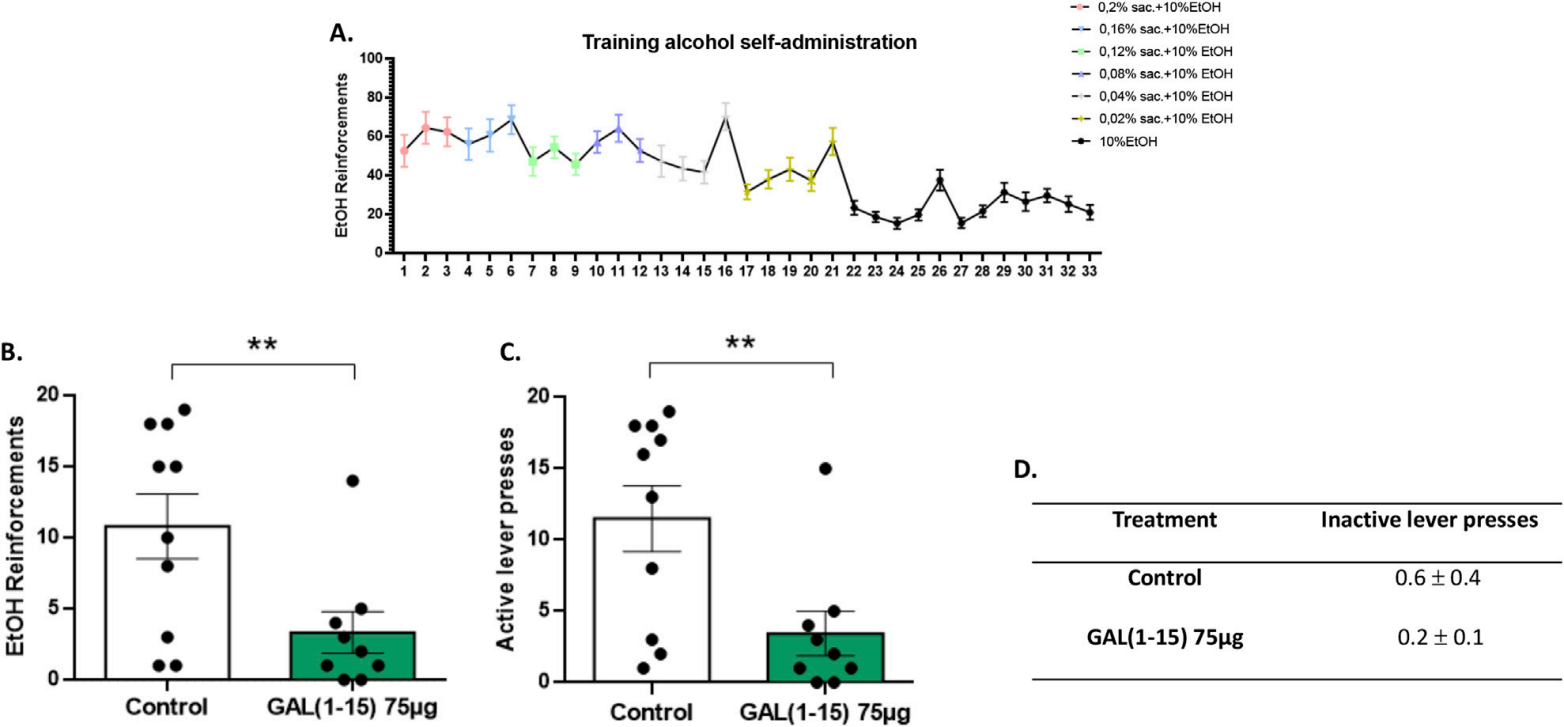
Effect of the intranasal administration of Galanin (1–15) [GAL (1–15)] on alcohol self-administration test. GAL (1–15) 75 µg (n = 9) or distilled water (n = 10) was injected intranasally 1 h before the test; rats received 10 µL of substance into each nostril. Animals injected with distilled water were considered the control group. **(A)** Progress of the animals during the training phase, illustrating the acquisition of alcohol self-administration across days. **(B,C)** Vertical bars represent a mean ± standard error of the mean number of reinforcements and active lever presses during the 30-min alcohol self-administration session. **p < 0.01 vs. the control group, according to a Student’s t-test. **(D)** Data represent the mean ± standard error of the mean of inactive lever presses during the test period. There are no differences between the experimental groups according to a Student’s t-test.

The animals initially received a saccharin–alcohol solution, and the saccharin concentration was gradually reduced over the training days until they self-administered 10% alcohol (Figure 3A).

Results demonstrated that intranasal administration of GAL (1–15) significantly reduced alcohol self-administration. Specifically, animals receiving GAL (1–15) exhibited a marked decrease in the number of alcohol reinforcements obtained during the self-administration session (Student’s t-test, t_17_ = 2.69, p < 0.01; Figure 3B). In addition, active lever presses, which indicate the motivation to obtain alcohol, were also significantly reduced following GAL (1–15) administration (Student’s t-test, t_17_ = 2.83, p < 0.01; Figure 3C). These findings suggest that GAL (1–15) effectively attenuates the drive to seek and consume alcohol when administered via the intranasal route.

Importantly, the observed reduction in alcohol self-administration was specific for this behavior, as the total number of inactive lever presses did not significantly differ between the GAL (1–15) and control groups (Figure 3D).

### 3.2 Toxicological results

#### 3.2.1 Cardiotoxicity assessment of GAL (1–15) in hERG-expressing HEK cells using the FluxOR™ potassium assay

**FIGURE 4:**
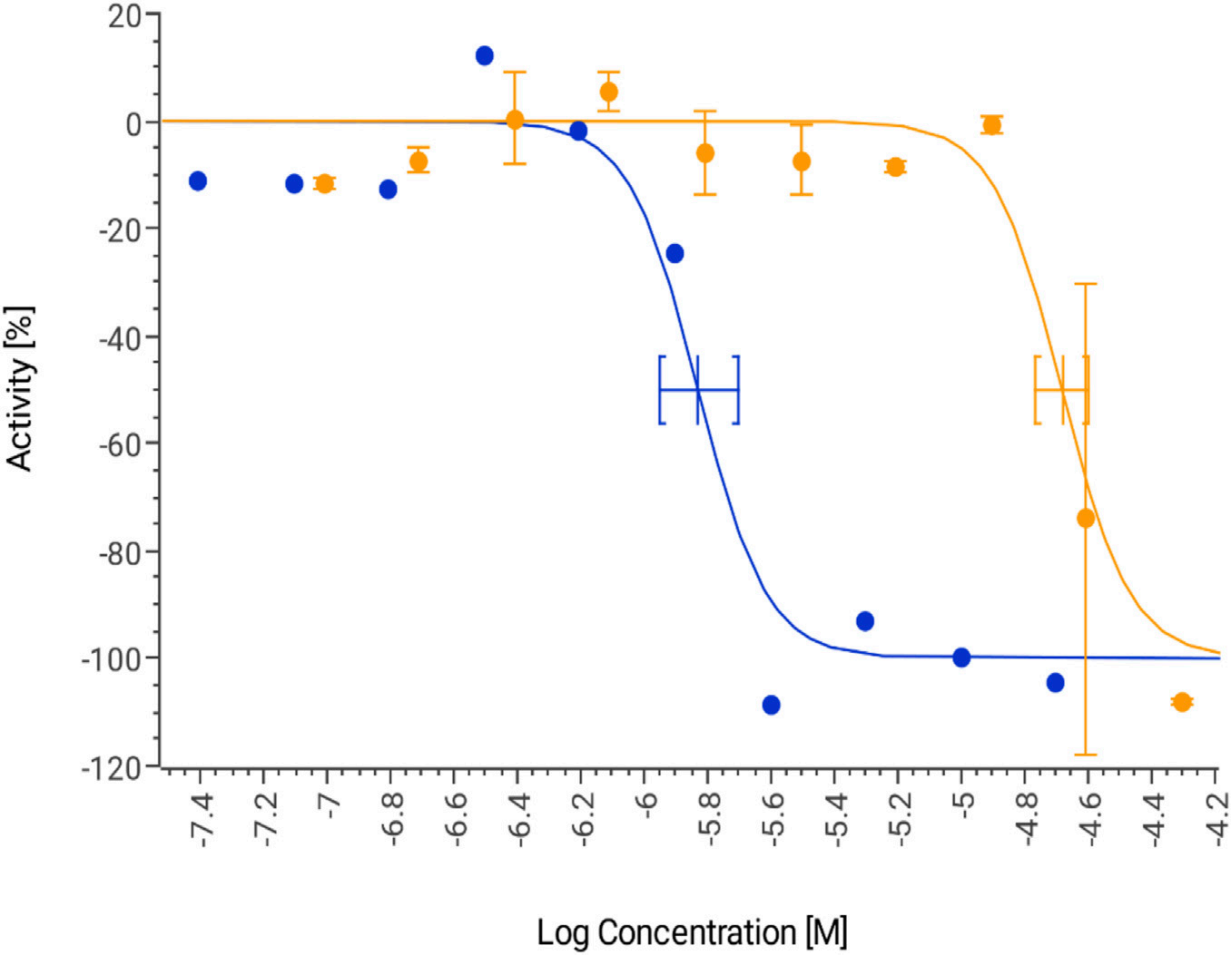
Dose response curve for GAL 1–15 (orange) and positive control astemizole (blue) tested for hERG inhibition. Data are expressed as the means ± S.D. (n = 3).

The potential cardiotoxicity of GAL (1–15) was evaluated through the inhibition of the hERG potassium channel, a critical factor in drug-induced cardiac arrhythmias. The assay was performed in HEK293 cells stably expressing the hERG channel, using the FluxOR™ potassium assay to determine the half-maximal inhibitory concentration (IC50). The results, presented in Figure 4, demonstrate that GAL (1–15) exhibited an IC50 value of 20.9 μM (95% confidence interval [CI]: 17.2–25.3 μM). In contrast, the positive control, astemizole, a well-characterized hERG inhibitor, displayed an IC50 of 0.39 μM (95% CI: 0.18–0.81 μM), consistent with its known cardiotoxic profile.

According to established classification criteria, compounds with an IC50 below 10 μM are considered cardiotoxic, while those with an IC50 above this threshold are classified as non-cardiotoxic (Annang et al., 2021). Based on these parameters, GAL (1–15) does not exhibit significant hERG inhibition and is therefore categorized as non-cardiotoxic under the experimental conditions used.

#### 3.2.2 Genotoxicity assessment of GAL (1–15) using the in vitro Micronucleus Test (MNT)

The potential genotoxicity of GAL (1–15) was evaluated using the *In Vitro* Micronucleus Test (MNT), a widely accepted assay for detecting chromosomal damage and genotoxic effects in cultured cells. The assay was performed following established guidelines, assessing micronucleus formation as a marker of DNA damage and chromosomal instability.

For this study, six independent wells were analyzed, with a minimum of 36 image fields per well captured at ×20 magnification to ensure robust statistical evaluation. The results, summarized in Table 1, indicate that GAL (1–15) did not induce micronucleus formation at any of the tested concentrations, with the highest concentration evaluated being 50 μM. No significant increase in micronucleated cells was observed compared to control conditions, suggesting the absence of genotoxic effects under the experimental parameters employed (Durcik et al., 2023; Cotman et al., 2023; Oecd, 2016).

**TABLE 1:** Results of compounds and positive control (CTR+) in MNT test without metabolic activation.

*Sample*	Concentration	No. binucleate cells	% Cytostasis	% MN (No. of binucleated cells with MN)
CTR-		**2,716**	-	**8.06 (217)**
GAL (1–15)	50 µM	3,108	0.1	7.94 (246)
GAL (1–15)	25 µM	3,135	0	8.70 (272)
GAL (1–15)	12.5 µM	3,209	0	8.53 (273)
GAL (1–15)	6.25 µM	3,210	0	8.28 (265)
GAL (1–15)	3.125 µM	3,324	0	8.39 (278)
CTR + (MitoC)	**1 µM**	**971**	39.4	27.8 (269)
CTR + (MitoC)	**0.5 µM**	**1,617**	14.76	19.91 (321)

GAL (1–15) was assayed at different concentrations and results are calculated in terms of number of binucleate cells, cytostasis percentage, and percentage of micronuclei in binucleate cells.

The controls are highlighted in bold.

#### 3.2.3 Cytochrome P450 inhibition assay and drug-drug interaction potential of GAL (1–15)

The potential for drug-drug interactions mediated by cytochrome P450 (CYP450) inhibition was assessed for GAL (1–15) using a standardized CYP450 inhibition assay. This assay evaluates the ability of a compound to inhibit key CYP450 isoforms involved in drug metabolism, which is critical for predicting possible drug interactions.

For assay quality control, the RZ’ factor, a widely used statistical measure of assay robustness and reliability, was determined. In all cases, this parameter demonstrated values equal to or greater than 0.70, confirming the high quality and reproducibility of the assay. Additionally, the IC50 values obtained for the control inhibitors corresponded closely to previously validated reference data, further ensuring the reliability of the assay results.

**FIGURE 5:**
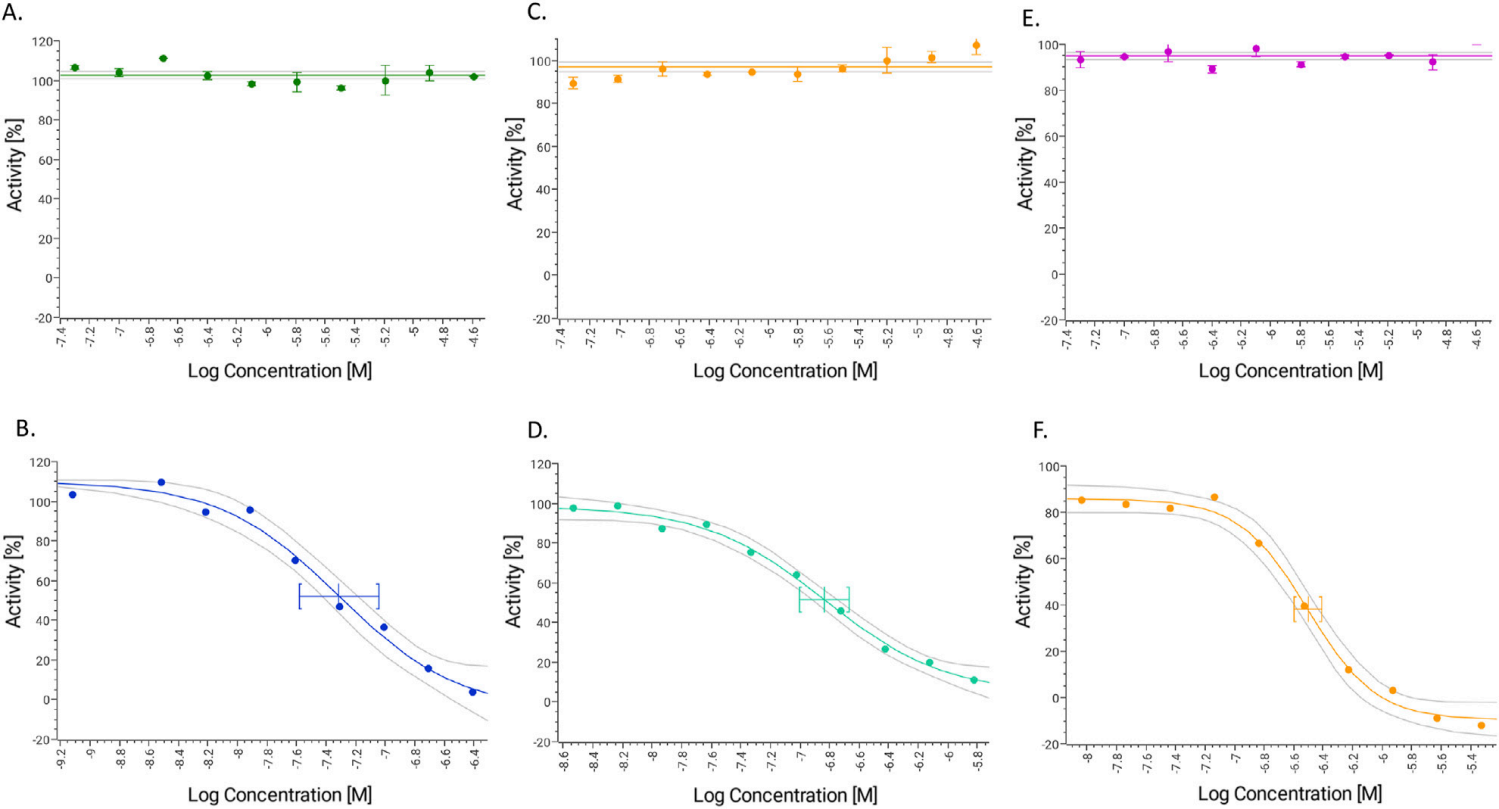
Dose response curve for GAL 1–15 and positive controls tested in three different isoforms of Cytochrome P450 **(A)** GAL 1–15 and **(B)** ketoconazole (CYP3A4). **(C)** GAL 1–15 and **(D)** Quinidine (CYP2D6). **(E)** GAL 1–15 and **(F)** sulfaphenazole (CYP2C9). (n = 3).

Based on established classification criteria, compounds are categorized into three groups based on their inhibitory potency: strong inhibitors (IC50 < 1 μM), moderate inhibitors (1 μM < IC50 < 10 μM), and weak inhibitors (IC50 > 10 μM). GAL (1–15) exhibited weak inhibition across all three CYP450 isoforms tested, with IC50 values exceeding 23 μM for each isoform (Figure 5; Table 2). These results indicate that GAL (1–15) has a low potential for CYP450-mediated drug-drug interactions, suggesting a favorable metabolic profile that reduces the likelihood of adverse interactions with co-administered drugs.

**TABLE 2:** IC50 values for GAL (1–15) inhibition assay tested CYP3A4, CYP2D6 and CYP2C9. (N = 3). N.a. not applicable (no inhibition detected).

Compound	CYP3A4 IC_50_ (µM) [lower and upper 95%]	CYP2D6 IC_50_ (µM) [lower and upper 95%]	CYP2C9 IC_50_ (µM) [lower and upper 95%]
GAL(1–15)	>26	>26	>26
Ketoconazole	0.049 [0.026–0.090]	N.a.	N.a.
Quinidine	N.a.	0.146 [0.099–0.215]	N.a.
Sulfaphenazole	N.a.	N.a.	0.313 [0.253–0.387]

## 4 Discussion

The results clearly demonstrate that intranasal administration of GAL (1–15), when used alone, produces a significant reduction in alcohol self-administratin, as shown in the operant self-administration model. These findings provide compelling evidence for the therapeutic potential of GAL (1–15) as a novel standalone intervention for alcohol use disorder. Additionally, while intranasal GAL (1–15) induces a prodepressive effect, prior research and our data support its efficacy as an adjunct to SSRIs for modulating depression-related behaviors, suggesting a dual therapeutic strategy that is tailored to the specific disorder.

Moreover, our findings further highlight the cardiac safety of GAL (1–15) and, importantly, GAL (1–15) exhibited no genotoxic potential at the tested concentrations, meeting the non-genotoxic classification criteria outlined in the experimental protocol. Also, GAL (1–15) is unlikely to interfere with CYP450 enzymatic activity at physiologically relevant concentrations, reinforcing its suitability for further drug development and clinical evaluation. Collectively, these results support the favorable safety profile of GAL (1–15) and its potential as a promising therapeutic candidate.

To investigate the potential depression-related effects of intranasal GAL (1–15), we evaluated its impact on behavioral despair using the FST, a widely accepted paradigm for assessing antidepressant- and depression-like behaviors in rodents. Previous studies demonstrated that intracerebroventricular administration of GAL (1–15) at 3 nmol, significantly increased immobility by 44% and decreased climbing behavior by 46%, with similar effects observed at 6 nmol, indicating a pronounced depression-like phenotype [Millón, 2014, A role for galanin N-terminal fragment (1–15) in anxiety- and depression-related behaviors in rats]. In the present study, intranasal GAL (1–15) produced a comparable dose-dependent response, with the 75 μg dose eliciting the most robust effects. Notably, these behavioral changes persisted 1 h post-administration, suggesting sustained modulation of mood-related behaviors. Given the translational relevance of intranasal drug delivery, these findings provide valuable insights into the neuromodulatory role of GAL (1–15) as a potential adjunct to SSRIs in modulating depression-related behavior and its potential implications for mood disorders.

In addition to its effects on depression-like behavior, intranasal GAL (1–15) also modulated alcohol self-administration, as evaluated using the operant self-administration model—a well-established paradigm for studying motivated drug-seeking behavior. In the present study, intranasal administration of GAL (1–15) led to a marked reduction in the number of alcohol reinforcements obtained during the self-administration sessions. This effect was accompanied by a significant decrease in active lever presses, indicating a specific reduction in alcohol motivation. Notably, the total number of inactive lever presses remained unchanged between GAL (1–15) and control groups, confirming that the observed reduction in alcohol reinforcement was not attributable to a general locomotor impairment or non-specific behavioral suppression but to a targeted decrease in alcohol motivation.

These results are consistent with previous findings from intracerebroventricular administration, GAL (1–15) at 3 nmol significantly reduced the number of alcohol reinforcements, indicating a decrease in alcohol-induced motivational drive (Cantero-García et al., 2022a). Similarly, GAL (1–15) has been shown to reduce motivation for both natural and artificial reinforcers, including saccharin self-administration (Millón et al., 2019a) and to decrease alcohol preference and consumption by 90% in a voluntary intake model (Millón et al., 2019b). Furthermore, combining GAL (1–15) with the antidepressant escitalopram (ESC) not only reduced alcohol self-administration but also mitigated ESC-induced adverse effects in depression-related behavioral tests (Cantero-García et al., 2022b).

Collectively, these findings underscore the therapeutic potential of GAL (1–15) in modulating both depression and in alcohol use disorder. In the present study, intranasal GAL (1–15) produced a comparable dose-dependent response, with the 75 μg dose eliciting the most robust effects. Notably, these behavioral changes persisted 1 h post-administration, suggesting sustained modulation of mood-related behaviors as a potential adjunct that potentiates SSRIs. - and addiction-related behaviors. The intranasal route ensures efficient drug delivery to the central nervous system and enhances the translational relevance of GAL (1–15) by aligning with clinically viable administration methods.

Although our findings suggest effective nose-to-brain delivery, direct evidence of central biodistribution is still lacking. Future studies should include quantitative LC–MS/MS analyses in relevant brain regions (e.g., olfactory bulb, prefrontal cortex, and hippocampus) or employ fluorescent analogues to visualise transport. Such experiments would provide more substantial support for the proposed nasal route of delivery.

The safety profile of GAL (1–15) was evaluated in several key areas. Regarding cardiotoxicity, GAL (1–15) was tested for its effect on the hERG potassium channel, which is associated with drug-induced arrhythmias, particularly QT interval prolongation. Although the link between QT prolongation and arrhythmias is still under study, hERG inhibition is a primary cause and a focus of safety assessments. Therefore, testing the interaction of a compound with the hERG potassium channel in heterologous expression systems is recommended by the International Conference on Harmonisation (ICH) as one of the non-clinical testing methods for assessing the potential of a test compound to prolong the QT interval. Using a FluxOR™ potassium assay in HEK293 cells expressing the hERG channel, GAL (1–15) did not significantly inhibit hERG, confirming its non-cardiotoxic profile under the tested conditions.

In the *In Vitro* Cell Micronucleus Test (MNT), GAL (1–15) did not induce micronucleus formation at concentrations up to 50 μM, suggesting no genotoxic effects. The MNT is a useful tool for genotoxicity testing because of its capacity to detect not only clastogenic and aneugenic events but also some epigenetic effects, and the protocol follows the recommendations of Test Guideline 487 (TG-487) of OECD guideline for the testing of chemicals (Durcik et al., 2023).

Additionally, GAL (1–15) was tested for potential drug-drug interactions via CYP450 inhibition. It showed weak inhibition across all three CYP450 isoforms tested, indicating a favorable metabolic profile with a reduced risk of adverse interactions when co-administered with other drugs. Prediction from *in vitro* studies is an integral part of early drug development, and drug agency guidelines (EMA, FDA and MHLW/PMDA). Computational models are increasingly used for quantitative prediction of *in vivo* interactions from *in vitro* experiments, and drug developers widely use these models to prepare for clinical trials (Hakkola et al., 2020). Our *in vitro* results provide excellent value for the following stages of study for the development as a GAL (1–15) drug.

The findings provide strong validation of the safety profile of GAL (1–15), indicating its promising potential for further pharmacological development. Nevertheless, to complement the results of this study, it would be interesting to address some aspects in the future, such as including females to increase the generalizability of the results, as well as evaluating the possible interaction with the carrier.

In conclusion, the demonstrated efficacy of intranasal GAL (1–15), combined with its favorable safety profile, positions GAL (1–15) as a promising candidate for advancing into more extensive clinical trials, allowing for a deeper understanding of its efficacy and long-term effects in therapeutic applications in treating mood disorders in combination with iSRS and substance use disorders.

## Data Availability

The raw data supporting the conclusions of this article will be made available by the authors, without undue reservation.
